# Bridging with Veno-Arterial Extracorporeal Membrane Oxygenation in Children: A 10-Year Single-Center Experience

**DOI:** 10.3390/life12091398

**Published:** 2022-09-08

**Authors:** Antonio Amodeo, Milena Stojanovic, Hitendu Dave, Robert Cesnjevar, Alexander Konetzka, Tugba Erdil, Oliver Kretschmar, Martin Schweiger

**Affiliations:** 1 Pediatric Cardiovascular Surgery, Pediatric Heart Center, Department of Surgery, University Children’s Hospital Zurich, 8032 Zurich, Switzerland; 2 Children’s Research Center, University Children’s Hospital Zurich, 8032 Zurich, Switzerland; 3 Pediatric Cardiology, Pediatric Heart Center, Department of Surgery, University Children’s Hospital Zurich, 8032 Zurich, Switzerland

**Keywords:** mechanical circulatory support, extracorporeal life support, ECMO, biomarkers, congenital heart disease, single ventricle, bridging

## Abstract

Veno-arterial extracorporeal membrane oxygenation (V-A ECMO) is frequently used in children with and without congenital heart disease (CHD). This study, of a single-center and retrospective design, evaluated the use and timing of V-A ECMO in a pediatric cohort who underwent V-A ECMO implantation between January 2009 and December 2019. The patients were divided into a pre-/non-surgical group and a post-surgical group. Among the investigated variables were age, gender, weight, duration of ECMO, ECMO indication, and ventricular physiology, with only the latter being statistically relevant between the two groups. A total of 111 children (58 male/53 female), with a median age of 87 days (IQR: 7–623) were supported using V-A ECMO. The pre-/non-surgical group consisted of 59 patients and the post-surgical group of 52 patients. Survival at discharge was 49% for the pre-/non-surgical group and 21% for the surgical group (*p* = 0.04). Single-ventricle physiology was significant for a worse outcome (*p* = 0.0193). Heart anatomy still has the biggest role in the outcomes of children on ECMO. Nevertheless, children with CHD can be successfully bridged with ECMO to cardiac operation.

## 1. Introduction

Extracorporeal membrane oxygenation (ECMO) is a form of mechanical circulatory support that can be offered to adults and children. Nevertheless, there are important differences to consider with particular reference to the type of ECMO circuit, implantation site, and indications. Traditionally, the aim of ECMO support is to bridge patients to recovery, decision, transplant, and other surgical treatments. This endpoint, due to the development of the patient’s condition, can even vary during the ECMO therapy itself. The use of ECMO in children has increased over the years because of improved technology, a better understanding of its physiology, and better management [1,2,3]. Regrettably, however, the mortality rate has remained unchanged at 46% [4], with 42% survival to discharge for neonates and 51% for pediatric patients according to the Extracorporeal Life Support Organization (ELSO) Report. Veno-arterial ECMO (V-A ECMO) is typically used in up to 97% of cases [4].

ECMO is now an established treatment pre-operatively and post-operatively. ECMO support is often used pre-operatively as a salvage procedure in patients with severe cardiogenic shock. Peri-operatively, weaning off cardiopulmonary bypass remains the most common indication. More recently, V-A ECMO has been used in both in- and out-of-hospital cardiac arrest [5,6].

ECMO is a suitable option for children with or without congenital heart disease (CHD) as a bridge to decision or further surgical treatment. This trend is supported by studies in children [7,8,9,10,11,12] and neonates [13,14]. Although ECMO is appropriate for patients with single-ventricle physiology following a bidirectional Glenn or Fontan procedure, the outcome remains worse compared to that of children with biventricular physiology [15,16,17,18]. Adverse events leading to significant complications and/or fatal outcomes occur regardless of the ECMO configurations and are multifactorial. The aim of this study was to discuss the outcome of V-A ECMO use in our pediatric patient population, with particular reference to the timing for surgical intervention.

## 2. Materials and Methods

This was a retrospective, single-center, observational study on the use of veno-arterial ECMO support at the University Children’s Hospital of Zurich, Switzerland. The study included patients less than 18 years of age who underwent ECMO support with the outcome measured at 30 days, 6 months, and 1 year. For the six-month and one-year follow-ups, we considered planned cardiological examinations after discharge from the index hospital stay.

The exclusion criteria were missing follow-up and therapy with a V-V (veno-venous) ECMO. If the patients underwent more than one ECMO run, only the first ECMO was taken into consideration to calculate the outcomes.

Two groups were defined. The “pre-/non-surgical group” consisted of patients who underwent ECMO insertion electively before surgery or as a bridge to decision, including those patients who did not undergo surgery. The “post-surgical group” included those patients who underwent V-A ECMO support within 30 days following a cardiac operation.

The outcomes of interest were survival at 30 days and at discharge, followed by survival at six months and one year. The following variables were considered: gender, age, weight at ECMO insertion, ECMO duration, ECMO indication, and heart physiology at the time of ECMO insertion.

While ECMO for intraoperative cardiac support can be offered to any child with CHD [19], some patients with anatomical or structurally abnormal hearts may still have high in-hospital mortality [20] and long-term morbidity [21] after ECMO. We conducted a subgroup analysis of patients suffering from CHD who underwent ECMO therapy, with the intent to find if ECMO can be a proper tool for bridging children with CHD to operation. Patients with CHD were identified via cardiac diagnosis by echocardiography.

### 2.1. ECMO Circuit and Implantation Techniques

Our standard approach consisted of the use of a console with a backup unit, Thoratec Centrimag^®^ or PediVas^®^ (Levitronix, Zurich, Switzerland), a heater unit, and a Sechrist 90 Air-Oxygen Mixer (Sechrist Industries, Anaheim, CA, USA). The circuit required approximately 250 mL of priming solution for children weighing up to 15 kg and flow ranges up to 1.7 L/min, whilst 750 mL was required for children above 15 kg and flow rates up to 7.0 L/min. Cannulation for neonates, infants, and small children was achieved either through the neck vessels or centrally via median sternotomy. We did not consider cannulation of the femoral vessels in small children in view of their inability to obtain full ECMO flow due to their small size. Neck vessel cannulation allows rapid deployment in emergency situations without interrupting chest compressions. A transverse skin incision was made in the lower third of the right sternocleidomastoid muscle. The internal jugular vein, the common carotid artery, and the vagal nerve were identified. Purse-string sutures were placed in the vein (V-V ECMO) and artery (V-A ECMO).

Cannulas were selected based on the weight of the patient and the desired achievable flow rate and were then connected to the ECMO circuit. A heparin bolus (50–100 IU/kg) was administered, independently of the cannulation site, before inserting the cannulas. Continuous heparin infusion was titrated using a goal-activated clotting time in the range of 160–180 s. The platelet count was maintained at a level of 100.000/mm^3^ or above. Peri-operative injection of one dose of prophylactic antibiotic (cefazolin) was administered during every ECMO implantation [22].

### 2.2. Statistical Analysis

Statistical analysis and presentation were conducted with the open-source R statistical software (version 4.1., R Foundation, Vienna, Austria). Any mention of significance refers to an alpha level of 5% if not otherwise stated, tested two-sided. The time until death was recorded from the day of the ECMO implantation until the one-year follow-up. The other variables, such as age, weight, and gender, were taken into account as uncontrolled covariates potentially biasing the outcome of the investigation. For the comparison of categorical variables, chi-square tests were used, while for continuous variables, *t*-tests were used. Multivariate Cox regression analysis was used to examine the relationship between all-cause mortality and the variables age, gender, group, weight, ECMO indication, and underlying heart physiology. A logistic regression analysis on survival as a binary outcome was modeled with the same variables. Hazard ratios (HRs) were estimated with the Cox proportional hazard model, including risk estimation and 95% confidence intervals (95% CIs). Kaplan–Meier curves were modeled with a comparison between both groups.

In a first step, the factorial response survival was analyzed with a logistic regression model as a function of the binary factors group (“pre-/non-surgical” or “post-surgical”), gender (“male” or “female”), cardiac (“failure“ or “no failure”), respiratory (“failure“ or “no failure”), and ventricular physiology (“biventricular” or “single-ventricle”) and the continuous covariates age, weight, and duration of ECMO. The linear model terms of the logistic regression model were augmented by multiway interactions in a step-wise manner, as required. All of the variables were checked for multicollinearity and residual outliers (Table A1). In the second step, the Kaplan–Meier model was computed with survival and time between ECMO implementation and the recorded day of death.

## 3. Results

### 3.1. Study Group

From January 2009 to December 2019, V-A ECMO was implanted in 111 patients, forming the study group, with a total of 53 female and 58 male patients. A good balance was found between both groups according to a simple chi-square test.

A total of 59 patients received ECMO either as a bridge to surgery or had no surgery and formed the pre-/non-surgical group, while 52 patients underwent ECMO therapy after cardiac surgery and formed the post-surgical group. The demographic data are summarized in Table 1.

The median age and weight were higher in the pre-/non-surgical group. The weight differed significantly, with a median of 6 kg (IQR = 15) in the pre-/non-surgical group, compared to 4 kg (IQR = 2) in the post-surgical group.

As shown, 70 of the 111 patients (63%) were suffering from CHD, 18 in the pre-/non-surgical group (30% of the group) and 52 in the surgical group (100% of the group). Among the 111 patients, 25 (22.5%) had single-ventricle physiology at the time of the ECMO implantation, with two in the pre-/non-surgical group (3.4% of the group) and 23 in the post-surgical group (44% of the group). Biventricular physiology was found in 86 of the 111 patients (77.4%), with 57 in the pre-/non-surgical group (96.6% of the group) and 29 in the post-surgical group (56% of the group).

The median duration of ECMO was four days overall, as well as in both groups (*p* = 0.13). Survival at 30 days was 52% and at discharge was 49% for the pre-/non-surgical group, while it was 31% at 30 days and 21% at discharge for the post-surgical group (*p* = 0.002). There was a total of 40 patients discharged alive (36%), 29 in the pre-/non-surgical group (49% of the group) and 11 in the post-surgical group (21% of the group). At the six-month and one-year follow-ups, all of the patients from both groups who left the hospital alive were still alive (Table 2).

**Figure 1:**
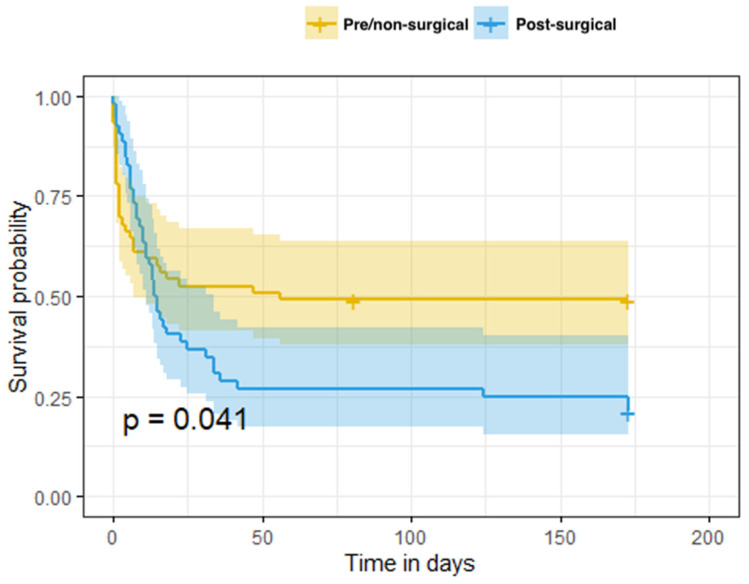
Kaplan–Meier model with survival and time between ECMO implantation and the recorded day of death.

The Kaplan–Meier model survival curves are shown in Figure 1. There was a significant difference in the survival rates between the two groups in the log-rank test. The median survival time was 14.5 days in the post-surgical group, compared to 56 days in the pre-/non-surgical group.

The Kaplan–Meier model results suggest that most deaths happened within 50 days of ECMO implantation. The estimated survival probability in the pre-/non-surgical group diminished rapidly in the first days to 50%, while in the post-surgical group it decreased to 25%.

In the multivariate Cox regression proportional hazard analysis, single-ventricle physiology was significant for worse outcomes (HR = 1.94, 95% CI = 1.04–3.61; *p* = 0.0365). The confidence interval of 1.04–3.61 before stratification for the hazard ratio shows that the associated increase in the risk of death might have only been relatively small. However, the *p*-value of 0.04 for all three overall tests (likelihood, Wald, and score) indicates that the model was significant. Therefore, the difference in the median survival time between the two groups can possibly be explained by the unequal distribution of single-ventricle and biventricular physiology, with 23 in the post-surgical group (44.2%) versus 2 in the pre-/non-surgical group (3.4%). In the statistical analysis, covariate age was not found to be significant and was dropped from the model, together with weight, cardiac failure, respiratory failure, and duration of ECMO.

Violations of the proportional hazard assumption for the variables of the group and underlying anatomy indicated the need for stratification to see if the significance of the difference held. A multivariate analysis with stratification in the anatomy variable still led to violations in the assumptions. An analysis with stratification in the group, however, found the difference in ventricular physiology to still be significant (Table 3).

There might be a higher risk for male patients, but the effect could only be estimated with a *p*-value of 0.051 in this study.

The estimated increase in risk for univentricular anatomy even increased slightly (HR = 2.09, 95% CI = 1.13–3.89; *p* = 0.0193).

In the post-surgical group, whether the timing from coming off bypass to ECMO may also be predictive of outcomes was analyzed. It was found that 25 patients in this group had ECMO on the day of the operation, 15 on the first post-operative day, and 12 on the second post-operative day; however, no correlation with the outcome (*p* > 0.3) could be observed.

### 3.2. Subgroup CHD Patients

In the whole population of this study, 70 patients were diagnosed with any form of CHD. In the pre-/non-surgical group, there were 18 patients with CHD, including 2 patients with single-ventricle physiology (Table 4).

The median ECMO duration time in this group was two days. While both patients with SV physiology died, the survival rate to discharge was 44% for those with biventricular physiology. Among these 18 patients in the pre-/non-surgical group, 10 had a cardiac operation after ECMO (55%), and, of these, 9 had a biventricular and 1 SV physiology. The median time from ECMO implantation to operation was five days. Seven of the operated patients were alive at one year, and one of the patients with biventricular physiology died during ECMO therapy. Another two patients died during their hospital stay after successful weaning off of ECMO, one (with SV) due to multiorgan failure after conversion to a ventricular assist device (VAD) and the other (with biventricular physiology) due to sepsis.

For the other eight CHD patients in the pre-surgical group, ECMO support was withdrawn due to complications of the ECMO itself or as the patients were not suitable for the necessary cardiac operation.

In the post-surgical group, 44% had single-ventricle physiology, while the majority (56%) had biventricular physiology. The median ECMO duration was four days. At one year, eight patients (28%) with biventricular and three (13%) with single-ventricular physiology were alive (Table 5).

### 3.3. Patients with More ECMO Runs

We decided not to take into consideration for the main analysis the second ECMO run on the same patient since we thought it could bring some confusion and bias. We found this approach already adopted in the literature [1].

In a short analysis, we found that in the pre-/non-surgical group, two patients had a second ECMO run, one of whom died in the hospital, and the other was alive at one year. In the post-surgical group, 13 patients had a second ECMO run; 2 were alive after one year, 2 were alive at 30 days but died before discharge, and 9 died in less than 30 days following their first ECMO implantation.

## 4. Conclusions

This study shows that children suffering from CHD can be successfully bridged to cardiac operation. Still, heart anatomy seems to have the biggest impact on the outcome. According to our data, children with single-ventricle physiology needing ECMO support, whether pre- or post-operatively, had the worst outcome.

If children after V-A ECMO support were discharged home, they had an excellent one-year survival rate. However, further long-term data on their outcome are needed.

Moreover, further studies are required to identify additional variables that may guide the timing, treatment optimization, and management of ECMO support.

## 5. Discussion

In this study, we analyzed our experience with children needing V-A ECMO support, focusing on the timing of ECMO installation. To better answer this question, we decided to divide the population into two groups depending on whether ECMO was installed before surgery (pre-surgical) or after surgery (port-surgical). It was important to include all patients who received V-A ECMO therapy so that children also receiving ECMO to whom cardiac surgery was never offered were included. This seemed important to answer the question of whether ECMO may be used as a bridge to the cardiac operation or not.

This selection process also explains the significant difference in the survival rates between the two groups. Seventy percent of the patients included in the pre-surgical group had no CHD, which was associated with better outcomes compared to the post-surgical patients. Of the 18 patients suffering from CHD, 55% were successfully bridged to cardiac operation using ECMO. Among these patients operated on, 70% were still alive at the one-year follow-up. In the remaining eight children on ECMO, the conditions were too poor for an operative solution and cardiac operation was, hence, not offered.

We think it is a justified approach to use ECMO as a bridge to decision in severely ill patients. ECMO treatment in this setting buys time to gather more information on prognostic factors (i.e., genetic analysis) to determine the outcome.

This study also confirmed the results of prior publications [23,24,25] in which univentricular physiology remained a predictor of poor outcomes in children on ECMO therapy with an HR of 2.09. Almost a quarter of all patients included had single-ventricle physiology. As expected, the distribution was quite unbalanced, with the majority in the post-surgical group; however, there were still patients (almost 4%) in the pre-surgical group who successfully bridged to cardiac operation. Heart anatomy still seems to have the biggest impact on outcomes. According to our data, children with single-ventricle physiology needing ECMO support, whether pre- or post-operatively, had the worst outcomes.

Other studies have successfully identified variables such as a high inotrope score, bleeding during ECMO, fluid overload, renal failure, initiation of ECMO in the intensive care unit, duration, weight, use and length of cardiopulmonary resuscitation, and pH or lactate before and during ECMO as prognostic markers [26,27,28,29,30]. In our cohort, some of these variables were investigated without showing significant differences between groups. In particular, weight, age, and duration of ECMO support were not associated with a worse outcome.

In the post-surgical group, only 2 of the 13 patients who underwent more than one ECMO run were alive at one year (15%). The outcomes of these patients are known to be poor [31]; however, focused studies with larger cohorts are required to validate these findings.

This study also tried to add mid-term data to the literature when reporting not only survival to discharge, but also survival at 6 and 12 months. Once children are discharged following ECMO support, they have excellent one-year survival rates regardless of diagnosis and/or prior surgical intervention. In this study, there were no deaths after hospital discharge until the one-year follow-up. Similar to others, we observed the highest mortality within the first days and only very few deaths after 30 days. In this population, only 7% died after the 30 day period. It would be interesting to investigate the long-term outcomes of children with CHD who undergo ECMO therapy since these outcomes (especially neurologic) are still poor [32].

In conclusion, we think that ECMO can be successfully used in children suffering from CHD, even with single-ventricle physiology, as a bridge to cardiac operation, but also as a bridge to decision, and even to abandon further therapy.

## 6. Limitations

All limitations of retrospective single-center studies, including the small number of patients in the cohort, apply here. Some factors, such as the time of ECMO deployment (day/night and weekday/weekend), and place of ECMO deployment (operation suite/intensive care unit), alongside the heterogeneity of the diagnosis, could not be properly included in the analysis or, therefore, properly researched as variables to foresee the outcomes.

The multivariate Cox regression proportional hazard analysis showed that the differences in the hazard ratios between the two groups can be explained by the different distribution of native physiology between the groups.

## Figures and Tables

**Table 1:** Demographics.

Variable	Total *n* = 111	Pre-/Non-Surgical *n* = 59	Post-Surgical *n* = 52	*p*-Value
Age in days Median (IQR)	87 (7–623)	137 (4–1670)	58 (8–125)	0.15
Male	58	31 (53%)	27 (52%)	0.051
Weight in kilograms Median (IQR)	4.5 (3–11)	6 (3–18)	4 (3–5)	0.028
Single-ventricle	25 (22.5%)	2 (3.4%)	23 (44%)	<0.001
Indication for ECMO Cardiac Respiratory	93 18	44 (75%) 15 (25%)	49 (94%) 3 (6%)	0.005 0.005

**Table 2:** Results.

	Total *n* = 111	Pre-/Non-Surgical *n* = 59	Post-Surgical *n* = 52	*p*-Value
Duration ECMO in daysMedian (IQR)	4 (2–6.5)	4 (2–6)	4 (3–7)	0.13
Survival at 30 days	47 (42%)	31 (52%)	16 (31%)	0.002
Survival at discharge	40 (36%)	29 (49%)	11 (21%)	0.002
Survival at 6 months	40 (36%)	29 (49%)	11 (21%)	0.002
Survival at 1 year	40 (36%)	29 (49%)	11 (21%)	0.002

**Table 3:** Analysis of the variables after stratification.

Variable	HR
Age	1.00 (1.00–1.00; *p* = 0.589)
Gender male	1.64 (1.00–2.68; *p* = 0.051)
Weight	0.97 (0.90–1.04; *p* = 0.398)
Univentricular	2.09 (1.13–3.89; *p* = 0.019)
Post-surgical group	1.10 (0.60–1.99; *p* = 0.764)

**Table 4:** CHD population in both groups.

Variable	CHD in Pre-/Non-Surgical *n* = 18	CHD in Post-Surgical *n* = 52
Single-ventricle	2 (11%)	23 (44%)
Biventricular	16 (89%)	29 (56%)
Cardiac operation Single-ventricle Biventricular	10 (55%) 1 (10%) 9 (90%)	52 (100%) 23 (44%) 29 (56%)
No cardiac operation	8 (44%)	0

**Table 5:** Results of subgroup CHD.

	CHD in Pre-/Non-Surgical *n* = 18	CHD in Post-Surgical *n* = 52
Duration of ECMO in days Median (IQR)	2 (1–5)	4 (3–7)
Survival at 1 year Single-ventricle Biventricular	0 7 (44%)	3 (13%) 8 (28%)

## Data Availability

All of the data of this study are in the Hospital-Database, available with the software “Phoenix^®^” of CGM Clinical (CompuGroup Medical Schweiz AG, Bern).

## Appendix A

**Table A1:** Logistic regression.

	Estimate	Standard Error	*z*-Value	Pr(>|z|)	Signif.
(intercept)	0.336	0.350	0.962	0.3359	
Gender = male	–0.629	0.428	–1.470	0.1417	
Ventricular = univ	–1.342	0.723	–1.857	0.0633	<0.1
Group = surgical	–0.857	0.476	–1.799	0.0720	<0.1

(Dispersion parameter for binomial family taken to be 1.) Null deviance: 145.1 on 110 degrees of freedom. Residual deviance: 130 on 107 degrees of freedom.
